# Cardio-metabolic risk factors and prehypertension in persons without diabetes, hypertension, and cardiovascular disease

**DOI:** 10.1186/1471-2458-13-730

**Published:** 2013-08-07

**Authors:** Peggy PC Chiang, Ecosse L Lamoureux, Anoop Shankar, E Shyong Tai, Tien Y Wong, Charumathi Sabanayagam

**Affiliations:** 1 Singapore Eye Research Institute, Singapore National Eye Centre, Level 6, 11 Third Hospital Avenue, Singapore 168751, Singapore; 2 Centre for Eye Research Australia, University of Melbourne, Melbourne, Australia; 3 Department of Ophthalmology, National University of Singapore, Singapore, Singapore; 4 Duke-NUS Graduate Medical School, Singapore, Singapore; 5 Department of Epidemiology, West Virginia School of Public Health, Morgantown, USA; 6 University Medicine Cluster, National University Health System, Singapore, Singapore

**Keywords:** Prehypertension, Metabolic syndrome, Indian, Cardiometabolic

## Abstract

**Background:**

Prehypertension has been shown to be an early risk factor of cardiovascular disease (CVD). We investigated the prevalence and pattern of cardiometabolic risk factors in prehypertension in three ethnic Asian populations in Singapore.

**Methods:**

We examined data from Chinese (n = 1177), Malay (n = 774), and Indian (n = 985) adults aged 40–80 years who participated in three independent population based studies conducted from 2004–2011 in Singapore who were free of diabetes, hypertension and previous CVD. Prehypertension was defined as systolic blood pressure (BP) 120–139 mm Hg or diastolic BP 80–89 mm Hg. Random blood glucose, glycated haemoglobin (HbA_1c_), body mass index (BMI), triglycerides, low-density lipoprotein (LDL) and high-density lipoprotein (HDL) cholesterol were examined as indicators of adverse cardiometabolic profile. The association between metabolic variables and prehypertension was examined using logistic regression models adjusting for potential confounders.

**Results:**

The prevalence of prehypertension was 59.8% (Chinese), 68.9% (Malays) and 57.7% Indians. Higher levels of blood glucose, HbA_1c_ and BMI were significantly associated with prehypertension in all three ethnic groups, odds ratio (95% confidence interval) of prehypertension in Chinese, Malays and Indians were: 1.42 (1.10, 1.83), 1.53 (1.05, 2.24), 1.49 (1.13, 1.98) for high-glucose; 3.50 (1.01, 12.18), 3.72 (1.29, 10.75), 2.79 (1.31, 5.94) for high-HbA_1c_; 1.86 (1.34, 2.56), 2.96 (2.10, 4.18), 1.68 (1.28, 2.20) for high-BMI. In addition, higher levels of LDL cholesterol in Chinese and higher levels of triglycerides were significantly associated with prehypertension. These associations persisted when metabolic variables were analysed as continuous variables.

**Conclusions:**

Higher levels of blood glucose, HbA_1c_ and BMI were associated with prehypertension in all three ethnic groups in Singapore. Screening for prehypertension and lifestyle modifications could potentially reduce the burden of CVD in otherwise healthy Asian adults living in Singapore.

## Background

Hypertension [1] is an important modifiable risk factor for cardiovascular disease (CVD). Prehypertension, an earlier stage in the continuum of hypertension where preventative efforts have been shown to be effective in delaying or preventing the onset of hypertension [2-4], is associated with increased future risk of hypertension [5], diabetes mellitus, and CVD [6]. Identifying and managing prehypertension have been recognized in national health policies as a priority to improve public health in some Western countries [7]. Recent studies using Western populations have shown that prehypertension is associated with adverse cardiometabolic risk profile even among apparently healthy populations [2,3].

To date no study has examined the association of cardiometabolic risk factors with prehypertension in Asia. This information is important because the prevalence of CVD is high among Asians and the contribution of major risk factors to CVD [8,9] and diabetes [10] have been shown to be different among Asians compared to Western populations. For instance, the onset of adverse clinical events occurs at a lower body mass index (BMI) level among Asians compared to Western populations [11]. Additionally, by further studying these associations separately among major Asian ethnic groups, a better understanding of the contribution of the key cardiometabolic factors to prehypertension in different ethnic groups can be more clearly elucidated.

Singapore has a diverse ethnic population of Chinese, Malay and Indians and therefore ideally placed to study the ‘Asian’ cardiometabolic risk profiles and its association with prehypertension. In the current study, we examined the association between cardiometabolic risk factors and prehypertension in an apparently healthy multi-ethnic Singaporeans without diabetes mellitus, hypertension and preexisting CVD. Additionally, we examined the association of non-metabolic variables including demographic, lifestyle and socioeconomic factors with prehypertension. Examining the contribution of both cardiometabolic and non-cardiometabolic factors to prehypertension in different ethnic groups may better inform about the pathogenesis of prehypertension and intervention programs aimed at preventing and stemming the progression of cardiac or metabolic illnesses.

## Methods

### Study design and procedure

We used data from three cross-sectional population based studies conducted in Singapore from 2004–2011: These studies include the Singapore Malay Eye (SIMES) Study [12] (n = 3280); the Singapore Indian Eye (SINDI) Study (n = 3400); [13] and the Singapore Chinese Eye (SCES) Study (n = 3353) [13]. These three studies examined the prevalence and impact of major eye diseases in ethnic Malays, Indians, and Chinese aged 40-80+ years residing in the South-Western part of Singapore (Singapore is a well-developed urban city). All three studies followed the same study protocol and were conducted in the same study clinic (Singapore Eye Research Institute, Singapore). Details of the study population and methods have already been published [12,13].

For each ethnicity, the cohort was selected based on an age-stratified random sampling strategy. In brief, in SiMES, 5600 individuals were selected by an age-stratified random sampling method from the computer generated random list of 16,069 Malay names provided by the Ministry of Home Affairs [12]. Of the 4,168 eligible individuals, 3280 participated in the study (78.7% response rate). In SINDI, 6,350 adults were selected by an age-stratified random sampling method from the computer generated random list of 11,616 Indian names provided by the Ministry of Home Affairs. Of the 4,497 eligible participants, 3,400 participated in the study (75.6% response rate). In SCES, 6,752 adults were selected by an age-stratified random sampling method from the computer generated random list of 12,000 Chinese names provided by the Ministry of Home Affairs. Of the 4,605 eligible participants, 3,353 participated in the study (72.8% response rate) [13].

The participation rates of eligible participants were 78.7% for Malays, 75.6% for Indians and 72.8% for Chinese. The final population sample was n = 10,033. We further excluded those with diabetes (n = 2207), hypertension (n = 4124) and pre-existing CVD (n = 87), missing information on variables included in the multivariable analysis (n = 36), leaving 2936 for the final analysis (Chinese = 1177, Malays = 774, Indians = 985). Questionnaires on demographic, lifestyle, personal and medical history were administered by trained interviewers. Physical examination by trained personnel included measurement of blood pressure and anthropometry. Information on random blood glucose, HbA_1c_, and lipids were obtained from non-fasting venous samples [12,13]. Written informed consent was obtained from all participants. The study adhered to the Declaration of Helsinki and ethics approval was obtained from the Singapore Eye Research Institute Institutional Review Board.

### Assessment of prehypertension

Systolic and diastolic blood pressures were measured by trained clinic research assistants using a digital automatic blood pressure monitor (Dinamap model Pro Series DP110X-RW, 100 V2; GE Medical Systems Information Technologies, Inc., Milwaukee, WI), after the subject was seated for at least 5 minutes. Blood pressure was measured twice, 5 minutes apart. A third measurement was made if the systolic blood pressure differed by more than 10 mmHg or the diastolic by more than 5 mmHg. The mean between the two closest readings was then taken as the blood pressure for that individual. The blood pressure monitor was regularly calibrated by trained clinic staff. Participants with history of diabetes, CVD, and hypertension (includes those on hypertensive medication(s)) at the time of the study were excluded from the statistical analysis. Prehypertension was defined as systolic blood pressure (BP) ranging from 120–139 mm Hg and/or diastolic BP ranging from 80–89 mm Hg (definition used by JNC7) [14].

### Assessment of cardiometabolic risk factors

We examined blood glucose, glycated haemoglobin (HbA_1c_), high-density lipoprotein (HDL), triglycerides, low density lipoprotein (LDL) and body mass index (BMI), as components of the metabolic syndrome. We defined the components of the metabolic syndrome categorically as 1. High-blood glucose, defined as blood glucose ≥ 5.5 mmol/L; 2. High-HbA_1c_ as hbA_1c_ > 6.5%; 3. Low-HDL cholesterol as HDL cholesterol < 1.0 in men and <1.3 mmol/L in women; 4. High-triglycerides as triglycerides ≥ 1.7 mmol/L; 5. High-LDL cholesterol ≥ 2.6 mmol/L; and 6. High-BMI as BMI ≥ 25.0 kg/m^2^. The above cut-off points were chosen based on the clinical cut points for adverse outcomes defined by the World Health Organization (WHO) and ATP-III [15,16].

### Assessment of non-metabolic risk factors

Age, sex, education, income, smoking and alcohol consumption were assessed as non-metabolic risk factors. A standardized questionnaire was administered by trained interviewers to obtain information on participant socio-demographics, educational attainment, and personal and medical history. Age was defined as the age at the time of examination. The non-metabolic risk categories for prehypertension were defined as: age over 60 years and above, male gender, primary or below education, income < 1000 SGD, current smoker, and ever consumption of alcohol (i.e. reported to have consumed alcohol at least once a week in the past).

### Statistical analysis

All statistical analyses were performed using STATA version 12.0 (StataCorp, College Station, Tex., USA). Characteristics of the study population were examined using proportions, or means, and standard deviation (SD) as appropriate for the variables. Metabolic variables were analysed both as categorical and continuous (per SD increase) variables. We examined the association of metabolic variables with prehypertension in two logistic regression models: model 1 adjusted for age and sex and model 2 adjusted additionally for income, education status, smoking, and alcohol consumption. We then examined the association of non-metabolic categorical variables with prehypertension in the same regression models. We performed the analysis separately for each ethnic group. Finally, in a separate analysis, we combined all three ethnic groups and in the whole population, we tested for interaction between metabolic variables and ethnicity by including cross-product interaction terms in the corresponding multivariable logistic regression models.

## Results

Overall characteristics of the study population without hypertension, diabetes, and self-reported CVD by ethnic groups are shown in Table 1. Chinese were more likely to be older, female, secondary/above educated, had lower levels of BMI, and LDL cholesterol; Malays were less likely to be female, had higher levels of systolic BP, and lower levels of triglycerides and plasma glucose; Indians had higher levels of BMI and lower levels of HDL-cholesterol (Table 1).

**Table 1:** Characteristics of the study population in three multi-ethnic cohorts (n = 2936)

**Variables**	**Chinese**	**Malays**	**Indians**	**P value**
Age ^β^ (years)	54.4 ± 7.4	51.5 ± 9.1	52.0 ± 7.5	< 0.001
Sex ^β^, female (%)	671 (43.2)	364 (23.4)	518 (33.4)	< 0.001
Education ^β^, (%)				
primary/below	474 (40.2)	435 (55.7)	443 (43.9)	< 0.001
secondary and above	706 (59.8)	346 (44.3)	566 (56.1)	
Systolic BP ^β^ (mm Hg)	121.4 ± 11.1	124.0 ± 10.1	120.8 ± 11.1	< 0.001
Diastolic BP (mm Hg)	73.1 ± 7.5	73.6 ± 7.2	73.8 ± 7.2	0.06
BMI ^β^ (kg/m^2^)	22.7 ± 3.3	24.7 ± 4.6	25.4 ± 4.4	< 0.001
HDL-cholesterol ^β^ (mmol/l)	1.4 ± 0.4	1.4 ± 0.3	1.1 ± .3	< 0.001
LDL-Cholesterol ^β^ (mmol/l)	3.4 ± 0.8	3.6 ± 1.0	3.6 ± 0.8	< 0.001
Triglycerides ^β^ (mmol/l)	1.8 ± 0.9	1.4 ± 0.1	1.7 ± 0.9	< 0.001
Plasma glucose ^β^ (mmol/l)	5.6 ± 1.2	5.2 ± 1.1	5.5 ± 1.2	0.001
HbA_1c_, (%)	5.7 ± 0.4	5.7 ± 0.5	5.7 ± 0.5	0.1834

Data are normally distributed and presented as means (standard deviation) or number (%).

^β^difference between the three races, p < 0.05.

The age-adjusted prevalence of prehypertension was 63.7% in Chinese, 42.4% in Malays and 61.4% in Indians. Within each ethnic group (Table 2), compared to people with no prehypertension, those with prehypertension were generally older, had a lower education level (except for Malays), consumed alcohol (except for Chinese) and had higher BMI levels. Participants with prehypertension within each ethnic group also presented with higher levels of triglycerides, blood glucose, and HbA_1c_.

**Table 2:** Characteristics of the study population by prehypertension status in three multi-ethnic cohorts (n = 2936)

	**Chinese**			**Malays**			**Indians**		
**Variable**	**Prehypertension absent**	**Prehypertension present**	**p value**^**α**^	**Prehypertension absent**	**Prehypertension present**	**p value**^**α**^	**Prehypertension absent**	**Prehypertension present**	**p value**^**α**^
	**(n = 473)**	**(n = 704)**		**(n = 241)**	**(n = 533)**		**(n = 417)**	**(n = 568)**	
Age ^β^ (years)	53.0 ± 6.5	55.3 ± 7.8	< 0.001	49.1 ± 8.0	52.6 ± 9.4	< 0.001	51.0 ± 7.1	52.8 ± 7.7	< 0.001
Sex ^β^, female (%)	313 (66.2)	358 (50.9)	< 0.001	120 (49.8)	244 (45.8)	0.300	243 (58.3)	275 (48.4)	0.002
Education ^β^, (%)									
primary/below	161 (34.0)	312 (44.3)	< 0.001	129 (53.5)	301 (56.5)	0.724	152 (36.5)	279 (49.1)	0.002
Secondary	228 (48.2)	319 (45.3)		106 (44.0)	221 (41.5)		189 (45.3)	219 (38.6)	
Tertiary	84 (17.8)	73 (10.4)		6 (2.5)	11 (2.1)		76 (18.2)	70 (12.3)	
Income level ^β^, (%)									
< SGD1000	127 (28.0)	243 (36.0)	0.015	120 (50)	282 (52.3)	0.497	138 (33.8)	225 (39.2)	0.171
≥ SGD1000- < SGD3000	201 (44.3)	277 (41.0)		107 (44.2)	216 (40.1)		174 (42.7)	235 (40.9)	
≥ SGD3000	126 (27.8)	156 (23.1)		15 (6.2)	41 (7.6)		96 (23.5)	114 (19.9)	
Smoking status									
Never	384 (80.8)	523 (74.2)	0.028	130 (53.7)	301 (55.8)	0.764	318 (74.8)	430 (73.6)	0.912
Past	39 (8.2)	75 (10.6)		32 (13.2)	74 (13.7)		35 (8.2)	50 (8.6)	
Current	52 (11.0)	107 (15.2)		80 (33.1)	164 (30.4)		72 (16.9)	104 (17.8)	
Alcohol consumption (%)	53 (11.2)	85 (12.1)	0.638	10 (4.2)	9 (1.7)	0.039	39 (9.1)	83 (14.2)	0.014
Systolic BP (mm Hg)	110.1 ± 6.9	128.9 ± 5.9	< 0.001	112.0 ± 6.2	129.4 ± 6.1	< 0.001	110.1 ± 6.8	128.6 ± 5.9	< 0.001
Diastolic BP ^β^ (mm Hg)	68.0 ± 5.8	76.5 ± 6.6	< 0.001	68.3 ± 5.0	75.9 ± 6.8	< 0.001	69.2 ± 5.2	77.2 ± 6.6	< 0.001
BMI ^β^ (kg/m^2^)	22.0 ± 3.1	23.2 ± 3.4	< 0.001	23.2 ± 4.2	25.4 ± 4.6	< 0.001	24.7 ± 4.3	25.9 ± 4.4	< 0.001
HDL-cholesterol ^β^ (mmol/l)	1.4 ± 0.4	1.4 ± 0.4	< 0.001	1.4 ± 0.3	1.4 ± 0.3	< 0.001	1.1 ± 0.3	1.1 ± 0.3	< 0.001
LDL-Cholesterol (mmol/l)	3.33 ± 0.8	3.47 ± 0.8	< 0.001	3.53 ± 0.8	3.67 ± 0.8	< 0.001	3.46 ± 1.0	3.59 ± 0.9	< 0.001
Triglycerides ^β^ (mmol/l)	1.7 ± 1.0	1.9 ± 0.9	< 0.001	1.2 ± 1.0	1.5 ± 1.2	< 0.001	1.6 ± 0.9	1.8 ± 1.0	< 0.001
Plasma glucose ^β^ (mmol/l)	5.4 ± 1.1	5.7 ± 1.2	< 0.001	5.0 ± 1.1	5.2 ± 1.1	< 0.001	5.4 ± 1.1	5.6 ± 1.3	< 0.001
HbA_1c_^β^, (%)	5.7 ± 0.3	5.8 ± 0.4	< 0.001	5.6 ± 0.4	5.8 ± 0.6	< 0.001	5.6 ± 0.4	5.8 ± 0.5	< 0.001

Data are normally distributed and presented as means (standard deviation) or number (%).

^α^ Significant differences within races, p < 0.05.

^β^ difference between the three races, p < 0.05.

Table 3 shows the categorical association of metabolic risk factors with prehypertension. In all three ethnic groups, high blood glucose, HbA_1c_, and BMI was significantly associated with prehypertension in age, sex-adjusted and the multivariable models. In addition, high LDL cholesterol in Chinese, and high triglyceride in Malays showed a significant positive association with prehypertension. In models testing for interaction in the whole population, there was no significant interaction in the association between metabolic variables and prehypertension by ethnicity (p-interaction by ethnicity were: high-blood glucose-p = 0.87; high-HbA_1c_-p = 0.84; low-LDL-p = 0.67; high-triglycerides-p = 0.12; high-LDL-p = 0.83; high-BMI-p = 0.06). In analyses examining metabolic variables as continuous variables (Table 4), the associations of blood glucose, HbA_1c_ and BMI persisted in all three ethnic groups. Similar to the categorical analysis, triglycerides in Malays showed a significant positive association with prehypertension.

**Table 3:** Association between metabolic variables (categorical) and prehypertension by race

**Chinese**		
**Metabolic variables **^**α**^	**Model 1a**	**Model 2b**
High-blood glucose,	**1.39 (1.09, 1.79)**	**1.42 (1.10, 1.83)**
High-HbA_1c_	**4.03 (1.17, 13.89)**	**3.50 (1.01, 12.18)**
Low-HDL	1.04 (0.81, 1.34)	1.09 (0.83, 1.42)
High-triglycerides	1.26 (0.99, 1.61)	1.26 (0.98, 1.63)
High-LDL	**1.56 (1.11, 2.19)**	**1.58 (1.11, 2.24)**
High-body mass index	**1.91 (1.40, 2.63)**	**1.86 (1.34, 2.56)**
**Malays**		
**Metabolic variables **^**α**^	**Model 1a**	**Model 2b**
High-Blood glucose,	**1.49 (1.02, 2.19)**	**1.53 (1.05, 2.24)**
High-HbA_1c_	**3.62 (1.26, 10.40)**	**3.72 (1.29, 10.75)**
Low-HDL	1.23 (0.84, 1.79)	1.24 (0.85, 1.82)
High-triglycerides	**2.09 (1.46, 2.99)**	**2.08 (1.45, 2.98)**
High-LDL	1.39 (0.92, 2.12)	1.40 (0.91, 2.13)
High-body mass index	**2.94 (2.09, 4.13)**	**2.96 (2.10, 4.18)**
**Indians**		
**Metabolic variables **^**α**^	**Model 1a**	**Model 2b**
High-Blood glucose,	**1.43 (1.08, 1.88)**	**1.49 (1.13, 1.98)**
High-HbA_1c_	**2.64 (1.29, 5.42)**	**2.79 (1.31, 5.94)**
Low-HDL	0.94 (0.72, 1.24)	1.00 (0.76, 1.33)
High-triglycerides	1.30 (0.99, 1.71)	1.33 (1.00, 1.76)
High-LDL	1.50 (0.99, 2.28)	1.40 (0.91,2.15)
High-body mass index	**1.69 (1.30, 2.20)**	**1.68 (1.28, 2.20)**

Please note that each metabolic variable are analysed as separate statistical models.

All data are presented in Odds Ratio (OR) and 95% confidence interval (CI) i.e. OR (95%CI).

a Adjusting for age and gender.

b Adjusting for age, gender, education status, income, smoking status, alcohol consumption.

^α^ Significant results are bolded.

**Table 4:** Association between metabolic variables (continuous, per standard deviation increase) and prehypertension by race

**Chinese**		
**Metabolic variables **^**α**^	**Model 1a**	**Model 2b**
Blood glucose, mmol/L	**1.18 (1.06, 1.32)**	**1.18 (1.06, 1.32)**
HbA_1c_,%	**1.58 (1.13, 2.21)**	**1.51 (1.07, 2.13)**
HDL mmol/L	0.88 (0.64, 1.22)	0.87 (0.62, 1.21)
Triglycerides, mmol/L	1.44 (0.99, 1.32)	1.14 (0.98, 1.32)
LDL, mmol/L	1.00 (1.00, 1.01)	1.00 (1.00, 1.01)
Body mass index, kg/m^2^	**1.12 (1.08, 1.16)**	**1.12 (1.08, 1.17)**
**Malays**		
**Metabolic variables **^**α**^	**Model 1a**	**Model 2b**
Blood glucose, mmol/L	**1.25 (1.07, 1.46)**	**1.27 (1.09, 1.49)**
HbA_1c_,%	**2.14 (1.49, 3.07)**	**2.17 (1.51, 3.11)**
HDL mmol/L	0.62 (0.38, 1.01)	0.62 (0.37, 1.02)
Triglycerides, mmol/L	**1.38 (1.18, 1.63)**	**1.38 (1.17, 1.62)**
LDL, mmol/L	1.00 (1.00, 1.01)	1.00 (1.00, 1.01)
Body mass index, kg/m^2^	**1.16 (1.11, 1.20)**	**1.15 (1.11, 1.20)**
**Indians**		
**Metabolic variables **^**α**^	**Model 1a**	**Model 2b**
Blood glucose, mmol/L	**1.17 (1.04, 1.30)**	**1.20 (1.07, 1.35)**
HbA_1c_,%	**1.76 (1.32, 2.34)**	**1.84 (1.36, 2.48)**
HDL mmol/L	0.87 (0.56, 1.35)	0.80 (0.50, 1.26)
Triglycerides, mmol/L	1.16 (1.00, 1.34)	1.16 (1.00, 1.35)
LDL, mmol/L	1.00 (1.00, 1.01)	1.00 (1.00, 1.01)
Body mass index, kg/m^2^	**1.09 (1.06, 1.13)**	**1.09 (1.05, 1.13)**

All data are presented in Odds Ratio (OR) and 95% confidence interval (CI) i.e. OR (95%CI).

a Adjusting for age and gender.

b Adjusting for age, gender, education status, income, smoking status, alcohol consumption.

^α^ Significant results are bolded.

When non-metabolic factors were examined (Table 5), those aged 60 years and above and being male were associated with prehypertension in all three ethnic groups. For Indians, primary or below education was positively associated with prehypertension.

**Table 5:** Association between non-metabolic variables (categorical) and prehypertension by race

**Chinese**		
**Metabolic variables **^**α**^	**Model 1a**	**Model 2b**
Age > 60 years	**2.20 (1.61, 3.01)**	**1.96 (1.41, 2.74)**
Sex, male	**1.87 (1.46, 2.38)**	**1.95 (1.45, 2.63)**
Current smoker (Yes)	1.00 (0.80, 1.24)	0.99 (0.79, 1.23)
Alcohol intake (Yes)	0.85 (0.58, 1.26)	0.83 (0.56, 1.25)
Education, primary or below	**1.40 (1.09, 1.79)**	1.26 (0.96, 1.64)
Income, < $SGD1000	**0.80 (0.67, 0.95)**	0.90 (0.74, 1.10)
**Malays**		
**Metabolic variables **^**α**^	**Model 1a**	**Model 2b**
Age > 60 years	**2.41 (1.46, 3.98)**	**3.01 (1.75, 5.18)**
Sex, male	1.10 (0.81, 1.50)	**1.63 (1.05, 2.55)**
Current smoker (Yes)	0.82 (0.62, 1.09)	0.79 (0.59, 1.06)
Alcohol intake (Yes)	0.41 (0.16, 1.03)	0.52 (0.20, 1.36)
Education, primary or below	0.96 (0.70, 1.32)	0.97 (0.69, 1.38)
Income, < $SGD1000	1.11 (0.85, 1.46)	1.11 (0.82, 1.49)
**Indians**		
**Metabolic variables **^**α**^	**Model 1a**	**Model 2b**
Age > 60 years	**1.84 (1.28, 2.65)**	**1.57 (1.07, 2.30)**
Sex, male	**1.48 (1.14, 1.91)**	**1.97 (1.39, 2.78)**
Current smoker (Yes)	0.81 (0.64, 1.03)	0.78 (0.60, 1.00)
Alcohol intake (Yes)	1.38 (0.91, 2.11)	1.54 (1.00, 2.37)
Education, primary or below	**1.70 (1.31, 2.22)**	**1.69 (1.27, 2.26)**
Income, < $SGD1000	**0.77 (0.64, 0.94)**	0.89 (0.71, 1.11)

All data are presented in Odds Ratio (OR) and 95% confidence interval (CI) i.e. OR (95%CI).

a Adjusting for age and gender.

b Adjusting for age, gender, education status, income, smoking status, alcohol consumption.

^α^ Significant results are bolded.

## Discussion

The current study analysing data from three large population-based studies involving Chinese, Malay, and Indian adults free of diabetes, hypertension, and CVD showed that higher levels of blood glucose, HbA_1c_, and BMI were associated with prehypertension in all three ethnic groups. High levels of LDL in Chinese and triglycerides in Malays were associated with prehypertension. These associations were independent of potential confounding factors including age, sex, education, smoking and alcohol consumption and were consistently present when metabolic variables were analyzed as continuous or categorical variables. There are several notable points in our findings. First, to our knowledge this is the first study that examined the contribution of adverse cardiometabolic risk factors in healthy Asian adults to prehypertension, an earlier stage where preventive efforts have been shown to be effective in delaying or preventing the onset of hypertension and cardiovascular outcomes. Second, the main determinants of prehypertension were metabolic factors that are also well-known risk factors for diabetes. Third, these risk factors were not entirely the same ones identified in other Asian populations. For instance, the main determinants of prehypertension in a Japanese population were BMI and hyperlipidemia [17].

Our study found that BMI was the consistent risk factor associated with prehypertension across the three ethnic groups particularly among Malays. This finding supports past research which showed that being overweight is an established risk factor for prehypertension [18-21]. In the current study, and previous National Health surveys [8,22], Malays have been found to have the highest BMI among the three races, and have been reported to be the most overweight, followed by Indians and Chinese [23]. Our finding of an association of BMI with prehypertension is consistent with other Asian population: a previous meta-analysis of 24 studies in China where overweight was associated with 62% increased risk of developing hypertension [24]. Inflammation has been postulated to deregulate blood pressure control leading to prehypertension in individuals with high BMI [3]. People who are overweight and obese exhibit central adiposity and have an expanded visceral adipose tissue compartment. The increasing visceral adipose tissue accumulation renders the visceral adipose tissue dysfunctional, resulting in altered adipose tissue secretions that manifest as enhanced systemic inflammation [25].

In addition to BMI, our study also found HbA_1c_ and blood glucose to be associated with high odds of prehypertension in all three ethnic groups. Studies have found that adults with a high BMI, in parallel with the enhanced systemic inflammation, tend to develop greater insulin resistance [2,26-29]. Having a large BMI leads to higher fasting serum insulin levels and, in some individuals, a high-normal level of glycosylated haemoglobin [2]. High insulin levels and glucose concentrations above 5.5 mmol/L are associated with enhanced CVD risk [26] and people with prehypertension have been found to have increased insulin resistance [29]. In the current study, we found that higher levels of LDL cholesterol in Chinese and higher levels of triglycerides to be associated with prehypertension consistent with previous studies that have reported associations between various lipid components and prehypertension [18,30,31]. The increased whole body adipose tissue burden has also been found to foster dyslipidemia [2].

It is interesting to note that the risk factors identified in our study were not entirely the same ones identified in other Asian populations. Specifically, though BMI was a common risk factor among the three ethnicities in our study, BMI was more prominent among the Chinese living in China [32,33] and the Japanese; [17] whereas glycemic biomarkers showed a more notable presence in this group of Asians. This alludes to a possible interplay between nature and nurture on variations in risk factor patterns between populations. Our findings also further support our argument of the need to study these associations separately among major Asian ethnic groups in order to gain a better understanding of the contribution of the key cardiometabolic factors to prehypertension in different ethnic groups. Apart from age and gender, primary or below education was found to be associated with prehypertension in Indians. Educational attainment was selected as one of the proxies of SES because it has been reported as a key determinant of health status, particularly cardiovascular conditions [34]. The finding that a lower level of education is associated with prehypertension is not surprising. For instance, in one study, non-Hispanic white men and women with less years of education were found to be at higher risk of developing hypertension compared with their more educated counterparts [35]. It is possible that people with higher education levels hold occupations that are more stable and usually comes with a higher income bracket than people with a lower education who are often in blue-collar roles. The higher income often equates to more security, access to better food and shelter, and optional life indulgences such as going on relaxing holidays.

Our study has several strengths. Our sample size is very large and it is the first study to document and examine the risk factors of prehypertension in a representative multi-ethnic Asian cohort. However, as our study design was cross-sectional rather than longitudinal, causal inferences were not able to be made and it was not possible to determine whether these patients eventually converted to hypertension in the presence of these risk factors. Future research could follow up these patients and investigate the temporal relationship between BMI, glucose, and lipids and prehypertension. In addition, as the data for this study were collected from three cross-sectional studies conducted in different years, it is possible that differences in the prevalence of risk factors for CVD might have influenced our results. However, reports from the Singapore 2004 and 2010 National Health Surveys suggest that except for overweight/obesity, the prevalence of metabolic risk factors did not vary significantly between the two time points (prevalence of hypertension was 24.9% vs. 23.5% in 2004 vs. 2010, high total blood cholesterol was 18.7% vs. 17.4%, diabetes was 8.2% vs. 11.3% and overweight/obesity was 32.5% vs. 40.1%). Therefore, we do not believe that the main study results would be different if they were conducted in the same period [8,22]. Finally, readers should keep in mind that our results may not be generalizable to Chinese, Indians, and Malays living in countries other than Singapore.

## Conclusion

In conclusion, in a study involving apparently healthy Chinese, Malay and Indian adults free of diabetes, hypertension and CVD, we found that higher levels of blood glucose, HbA_1c_ and BMI were significantly associated with prehypertension in all three ethnic groups. Our findings suggest that prehypertension could serve as an early marker of adverse cardiometabolic profile in apparently healthy Asian adults and early detection and control of prehypertension could potentially reduce the burden of CVD in this Asian population.

## Competing interests

The authors declare that they have no competing interests.

## Pre-publication history

The pre-publication history for this paper can be accessed here:

http://www.biomedcentral.com/1471-2458/13/730/prepub
